# Association of Postoperative Infections After Fractures With Long-term Income Among Adults

**DOI:** 10.1001/jamanetworkopen.2021.6673

**Published:** 2021-04-19

**Authors:** Nathan N. O’Hara, C. Daniel Mullins, Gerard P. Slobogean, Anthony D. Harris, Dionne S. Kringos, Niek S. Klazinga

**Affiliations:** 1 Department of Orthopaedics, University of Maryland School of Medicine, Baltimore; 2 Department of Public and Occupational Health, Amsterdam Universitair Medische Centra, University of Amsterdam, Amsterdam Public Health Research Institute, Amsterdam, the Netherlands; 3 Department of Pharmaceutical Health Services Research, University of Maryland School of Pharmacy, Baltimore; 4 Department of Epidemiology and Public Health, University of Maryland School of Medicine, Baltimore

## Abstract

**Importance:**

Postoperative infections after a fracture exert tremendous costs on the health care system. However, the patient economic burden associated with a postoperative infection is unclear.

**Objective:**

To evaluate the association between a postoperative infection and long-term income among patients with surgically treated fractures.

**Design, Setting, and Participants:**

A retrospective cohort study linked academic trauma center data with Maryland tax records using inverse probability of treatment weighting to estimate between-group differences among 11 673 adults who underwent surgery to treat fractures of the extremities or pelvis between January 1, 2003, and December 31, 2016. Statistical analysis was performed from November 5, 2019, to August 30, 2020.

**Exposure:**

A postoperative infection within 1 year of injury.

**Main Outcomes and Measures:**

The primary outcome was the annual household income up to 6 years after injury. Household income incorporates multiple types of income, including wage earnings, taxable Social Security benefits, workers’ compensation, and disability benefits. Secondary outcomes included individual earnings, Social Security benefits, unemployment benefits, and catastrophic income loss.

**Results:**

The study included 11 673 patients (7756 male patients [66.4%]; mean [SD] age, 45.2 [19.2] years) with a mean (SD) preinjury household income of $30 505 ($89 030). A total of 403 patients (3.5%) had a postoperative infection. Postoperative infections were associated with a $6080 annual decrease (95% CI, −$12 114 to −$47; *P* = .048) in household income in the 6 years after injury. Postoperative infections were associated with a 6.6% increase (95% CI, 4.9%-8.3%; *P* < .001) in the risk of catastrophic wage loss within 2 years of the fracture and were associated with a 45% increase in the odds of receiving Social Security benefits (odds ratio, 1.45; 95% CI, 1.25-1.68; *P* < .001). However, incurring a postoperative infection was not associated with an increase in the value of the Social Security benefits received.

**Conclusions and Relevance:**

This study suggests that postoperative infections have significant and sustained income-associated implications for patients who experience a fracture. Current Social Security mechanisms may not offset the decreased income.

## Key Points

**Question:**

Is a postoperative infection after a fracture associated with long-term income loss?

**Findings:**

This cohort study of 11 673 patients that linked 14 years of academic trauma center data with state tax records estimated that, among patients with fractures treated surgically, a postoperative infection was associated with a $6080 decrease in annual household income in the 6 years after injury.

**Meaning:**

This study suggests that postoperative infections have significant and sustained income-associated implications for patients with a fracture.

## Teaser

This cohort study uses state tax records to evaluate the association between a postoperative infection and long-term income among patients with surgically treated fractures.

## Introduction

Postoperative infections exert a tremendous cost and resource burden on the health care system.^1,2,3^ Patients with postoperative infections require additional treatment, more diagnostic testing, readmission to the hospital or prolonged hospital stay, and other additional uses of scarce health care resources.^4,5^ The impact of postoperative infections also likely manifests more broadly in the economy through lost capacity and productivity of patients and their caregivers.^6,7^ Many postoperative infections can be systematically prevented through improved health care quality and infection prevention policies.^8,9^ Previous studies have demonstrated that the cost of infection prevention is typically much lower than the cost of treating the complication.^3,4,8,9,10^ However, there is a clear gap in knowledge on the long-term association of a postoperative infection with the patient’s economic well-being.^9^

Postoperative infections are common after the surgical treatment of a fracture. Soft tissue damage adjacent to the fracture and a systematic inflammatory response from the trauma are associated with a risk of postoperative infection ranging from 2% to 5% for most closed fractures to more than 20% for some types of open fractures.^11,12,13^ Fractures commonly occur during individuals’ most economically productive years and are associated with significant income loss.^6,7,12,14,15^ Adverse events after a fracture likely bear further economic consequences to the patient and society.

Tax records provide precise annual financial data to estimate the economic impact of patients’ medical conditions, treatments, and complications. However, health researchers rarely use tax records for medical research given strict privacy restrictions. The granularity of tax data enables the estimation of changes in the composition of earnings and social welfare benefits associated with a medical episode.

With unique access to state tax records, we aimed to estimate the association between a postoperative infection and the long-term income of patients who sustained a fracture. We hypothesized that a postoperative infection would decrease patients’ overall household income and increase Social Security benefits. Second, we explored variation in the relative association of postoperative infection with patient income based on policy-relevant subgroups. We hypothesized that a postoperative infection would be associated with greater relative income loss for patients with fracture who were more severely injured, lacked health insurance, and lived in an area of high deprivation.

## Methods

### Study Design

In this retrospective cohort study, we linked longitudinal data from annual state of Maryland tax records to individual-level hospital data from an academic trauma center in Baltimore, Maryland. The study used the target trial emulation approach, which included inverse probability of treatment weighting, for inference in observational research.^16^ The target trial emulation approach is particularly applicable for research questions such as this, where the exposure of interest cannot ethically be randomly assigned. The approach requires precise specification of the exposure groups to ensure positivity (all patients could have the exposure) and analytical techniques for conditional exchangeability (independence between the exposure and outcome).^17^ The University of Maryland, Baltimore institutional review board approved the study, including a waiver of informed consent per 45 CFR 46.116(d). This study followed the Strengthening the Reporting of Observational Studies in Epidemiology (STROBE) reporting guideline.

### Study Participants

We used *Current Procedural Terminology* (*CPT*) codes to identify adults who underwent surgery to treat a fracture of the extremities or pelvis between January 1, 2003, and December 31, 2016. We excluded patients admitted for a subsequent fracture during the study period and patients with a severe traumatic brain injury or spinal cord injury, defined as an Abbreviated Injury Scale score of 5 or more in those body regions.^18,19^ Patients without Social Security numbers recorded in their hospital billing records were also excluded.

Using the Social Security numbers obtained from hospital billing records, we linked hospital data with the patient’s state tax filings from 2000 through 2018. All eligible patients were matched to at least 1 tax record during the study period. However, the proportion of individuals who filed taxes varied over time (eFigure 1 in the Supplement).^20^

### Assessment of Exposure

The primary exposure was a postoperative infection defined using the Centers for Disease Control and Prevention (CDC) National Healthcare Safety Network criteria for a deep or organ space surgical site infection.^21^ Deep or organ space surgical site infections are more severe than superficial incisional surgical site infections and typically require additional surgical treatment. We modified the CDC’s 90-day window to include infections that occurred up to 12 months after surgery. This modification was based on evidence, commonly applied in fracture research, that nearly half of fracture-related infections occur more than 90 days from the index procedure.^22,23,24^ We also included infections that occurred at the site of amputation. All suspected infections were identified using the *CPT* codes, *International Classification of Diseases, Ninth Revision* codes, and *International Statistical Classification of Diseases and Related Health Problems, Tenth Revision* codes (eTable 1 in the Supplement). The medical record of each patient suspected to have a postoperative infection was independently reviewed by a certified infection preventionist to confirm the diagnosis at the fracture location.

### Study Outcomes

The primary outcome was household earnings up to 6 years after injury, calculated as tax-reported annual adjusted federal gross income. Adjusted gross income incorporates multiple types of income, including wage earnings, tax-exempt interest income, taxable Social Security benefits, workers’ compensation, and disability benefits. Consistent with the methods of Chetty et al,^25,26,27^ we recoded patients who did not file taxes and patients with negative incomes as having zero income for that specific year. Several components of household income were analyzed as secondary outcomes, including individual earnings and Social Security benefits. Social Security benefits included Retirement Income, Disability Insurance, and Supplemental Security Income. Receipt of Social Security benefits or unemployment insurance were binary secondary end points. Catastrophic income loss was also included as a secondary outcome. Consistent with prior research, we defined catastrophic income loss as mean wage earnings in the year of the injury plus 2 years after injury that was 50% less than the mean wage earnings in the 2 years before injury.^28^ All income values were adjusted to 2018 US dollars using the Consumer Price Index; this adjustment mitigates macroeconomic associations, such as the 2009 economic recession. Time zero was defined as the calendar year of the index fracture admission.

### Statistical Analysis

Statistical analysis was performed from November 5, 2019, to August 30, 2020. Patient characteristics were described using counts with proportions and mean (SD) values. We compared categorical data between the postoperative infection group and the control group using χ^2^ tests. Continuous data were compared using *t* tests.

We used inverse probability of treatment weights derived from the hospital and tax data to account for potential imbalance in factors prognostic of the exposure. Inverse probability of treatment weights form a pseudopopulation based on the conditional probability of a postoperative infection given the observed sociodemographic, economic, and medical data.^17,29^ We created the pseudopopulation by weighting each patient by the inverse of the conditional probability of receiving the exposure they did receive. Under this approach, the models estimate the mean difference between the potential outcomes if all patients did or did not have a postoperative infection. Demographic data used for the conditional probabilities included age, sex, race/ethnicity, the type of health insurance based on the National Health Interview Survey coding, and the Area Deprivation Index as a neighborhood-based measure of socioeconomic status at the time of injury.^30,31^ Clinical data included the mechanism of injury, location of the fracture, the number of surgical procedures associated with the index injury, and the Abbreviated Injury Scale score—an anatomically based injury severity scoring system—for all body regions. Comorbidity data included tobacco use, obesity, hypertension, diabetes, alcohol use, drug use, and kidney disease. In addition, we included the components of the tax-reported earnings for the 3 years prior to injury, to account for preinjury earning trends, and the calendar year of injury, to control for macroeconomic changes.

Inverse probability of treatment–weighted mixed-effects models were used to estimate the difference in income and catastrophic income loss associated with a postoperative infection. We fit distinct models censored at 1 year after injury until the proportion of tax filers was less than 50% of preinjury levels to evaluate the duration of any observed differences. We observed this level of tax filing attrition at 6 years after injury (eFigure 1 in the Supplement). The estimated differences in the models were interpreted as the annual mean difference in income during the censored time period. As a sensitivity analysis for the catastrophic income loss end point, we varied the threshold for wage loss from 25% to 75% of preinjury income and the window for wage loss from 2 to 5 years.

We analyzed heterogeneity in the association between a postoperative infection and household income and if the patient received Social Security benefits at 2 and 5 years after the fracture within several important clinical and policy subgroups. The subgroups included age (≥65 years vs <65 years), sex, race/ethnicity (White patients vs minority patients [including African American, Hispanic, American Indian or Alaska Native, Asian, Hawaiian Pacific Islander, or multiple races]), preinjury income quartile, neighborhood deprivation quartile, fracture severity (open vs closed fracture), fracture location, and health insurance status (uninsured vs some form of insurance). Neighborhood deprivation was calculated based on the Area Deprivation Index of the patient’s tax filing address in the year prior to injury. For the subgroup analyses, household income was transformed with a logarithm plus 1 to account for the right-skewed distribution and modeled using mixed-effects regression. The estimates can be interpreted as the postoperative infection’s relative association with household earnings within 2 and 5 years after injury. We estimated a postoperative infection’s relative association with obtaining Social Security benefits with generalized linear regression models with a binomial distribution reported as odds ratios (ORs) at 2 and 5 years after injury. All subgroup analyses used inverse probability of treatment weighting.

Missing covariate data were imputed using multiple imputations (eTable 2 in the Supplement).^32^ All *P* values were from 2-sided tests, and results were deemed statistically significant at *P* < .05. Owing to the increased potential for type I error with multiple comparisons, the estimates for secondary outcomes and the subgroup analyses should be interpreted as exploratory. All statistical analyses were performed with R, version 4.0.0 (R Group for Statistical Computing).

## Results

The study included 11 673 patients (7756 male patients [66.4%] and 3917 female patients [33.6%]; mean [SD] age, 45.2 [19.2] years) treated surgically for a fracture of the extremities or pelvis from January 1, 2003, through December 31, 2016 (Table 1; eFigure 2 in the Supplement). In the year prior to injury, the patients’ mean (SD) household income was $30 505 ($89 030). In the year before the injury, 1004 of 11 673 patients (8.6%) received Social Security benefits, and 385 of 11 673 (3.3%) received unemployment insurance. A total of 403 patients (3.5%) in the sample had a surgical site infection within 1 year of their index fracture fixation. There were notable differences between patients who developed a postoperative infection and the uninfected control patients. Specifically, patients with a postoperative infection were younger (mean [SD] age, 41.4 [14.4] years vs 45.3 [19.3] years; *P* < .001) and more likely to be male (310 [76.9%] vs 7446 [66.1%]; *P* < .001) and have an open fracture (321 [79.7%] vs 3119 [27.7%]; *P* < .001).

**Table 1.** Patient Characteristics

Characteristic	Patients No. (%)			*P* value
	Postoperative infection (n = 403)	Uninfected control (n = 11 270)	Overall (N = 11 673)	
Age, mean (SD), y	41.4 (14.4)	45.3 (19.3)	45.2 (19.2)	<.001
Sex				
Male	310 (76.9)	7446 (66.1)	7756 (66.4)	<.001
Female	93 (23.1)	3824 (33.9)	3917 (33.6)	
Race/ethnicity				
White	254 (63.0)	7496 (66.5)	7750 (66.4)	.70
African American	121 (30.0)	3005 (26.7)	3126 (26.8)	
Hispanic	6 (1.5)	135 (1.2)	141 (1.2)	
Other or unknown^a^	22 (5.5)	634 (5.6)	656 (5.6)	
Neighborhood deprivation				
Least deprivation quartile (ADI, 1-3)	81 (20.1)	2284 (20.3)	2365 (20.3)	.88
Second quartile (ADI, 4-6)	76 (18.9)	2289 (20.3)	2365 (20.3)	
Third quartile (ADI, 7-8)	86 (21.3)	2279 (20.2)	2365 (20.3)	
Most deprivation quartile (ADI, 9-10)	83 (20.6)	2281 (20.2)	2364 (20.3)	
Missing	77 (19.1)	2137 (19.0)	2214 (19.0)	
Mechanism of injury				
Motor vehicle accident	283 (70.2)	6627 (58.8)	6910 (59.2)	<.001
Fall	65 (16.1)	3059 (27.1)	3124 (26.8)	
Firearm	15 (3.7)	536 (4.8)	551 (4.7)	
Struck	7 (1.7)	323 (2.9)	330 (2.8)	
Cyclist	10 (2.5)	272 (2.4)	282 (2.4)	
Machinery	10 (2.5)	198 (1.8)	208 (1.8)	
Other	13 (3.2)	255 (2.3)	268 (2.3)	
Injury type				
Blunt	365 (90.6)	10 452 (92.7)	10 817 (92.7)	<.001
Penetrating	22 (5.5)	648 (5.7)	670 (5.7)	
Crush	16 (4.0)	156 (1.4)	172 (1.5)	
Other	0	14 (0.1)	14 (0.1)	
Glasgow Coma Scale score, mean (SD)	12.7 (4.0)	14.2 (2.5)	14.1 (2.5)	<.001
Health insurance				
Private employer based	140 (34.7)	3863 (34.3)	4003 (34.3)	<.001
Medicare	88 (21.8)	2298 (20.4)	2386 (20.4)	
Medicaid	101 (25.1)	2058 (18.3)	2159 (18.5)	
Uninsured	29 (7.2)	1447 (12.8)	1476 (12.6)	
Direct purchase	21 (5.2)	1160 (10.3)	1181 (10.1)	
Other public insurance	18 (4.5)	285 (2.5)	303 (2.6)	
Tricare, VA, or Champ	6 (1.5)	159 (1.4)	165 (1.4)	
Abbreviated Injury Scale score, mean (SD)				
Lower extremity	1.98 (0.93)	1.85 (1.08)	1.86 (1.07)	<.01
Upper extremity	0.94 (1.08)	0.98 (1.07)	0.98 (1.07)	.51
Abdominal	0.74 (1.13)	0.42 (0.87)	0.43 (0.88)	<.001
Face	0.36 (0.63)	0.36 (0.62)	0.36 (0.62)	.97
Head	0.90 (1.19)	0.67 (1.01)	0.67 (1.02)	<.001
Neck	0.11 (0.50)	0.08 (0.43)	0.08 (0.43)	.29
Spine	0.55 (0.93)	0.40 (0.85)	0.41 (0.85)	.01
Chest	0.96 (1.38)	0.69 (1.22)	0.70 (1.23)	<.001
Comorbidities				
Alcohol dependence	39 (9.7)	940 (8.3)	979 (8.4)	.34
Cancer	6 (1.5)	357 (3.2)	363 (3.1)	.06
Depression	29 (7.2)	755 (6.7)	784 (6.7)	.70
Diabetes	40 (9.9)	1075 (9.5)	1115 (9.6)	.80
Intravenous drug use	8 (2.0)	185 (1.6)	193 (1.7)	.60
Nonintravenous drug use	33 (8.2)	872 (7.7)	905 (7.8)	.74
Hypertension	67 (16.6)	2710 (24.0)	2777 (23.8)	<.001
Tobacco use	109 (27.0)	3433 (30.5)	3542 (30.3)	.14
Fracture location				
Humerus, clavicle, or scapula	57 (14.1)	1487 (13.2)	1544 (13.2)	.58
Radius or ulna	89 (22.1)	2622 (23.3)	2711 (23.2)	.58
Femur	134 (33.3)	2785 (24.7)	2919 (25.0)	<.001
Tibia or fibula	242 (60.0)	3976 (35.3)	4218 (36.1)	<.001
Pelvis or acetabulum	97 (24.1)	1842 (16.3)	1939 (16.6)	<.001
Hand	54 (13.4)	1162 (10.3)	1216 (10.4)	.046
Foot	56 (13.9)	998 (8.9)	1054 (9.0)	<.001
Open fracture	321 (79.7)	3119 (27.7)	3440 (29.5)	<.001

Abbreviations: ADI, Area Deprivation Index; VA, Veterans Affairs.

^a^ Other includes American Indian or Alaska Native, Asian, Hawaiian Pacific Islander, or multiple races.

### Household Income

**Figure 1.**
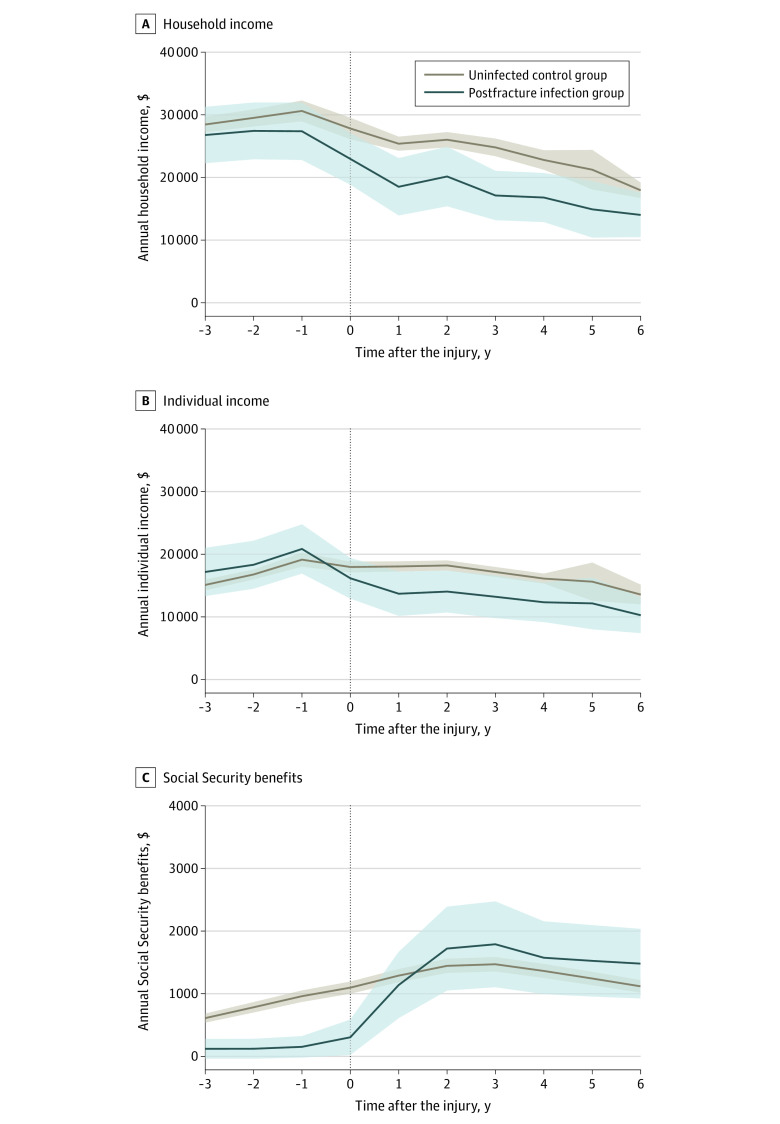
Association of Postoperative Infection With Household Income, Individual Income, and Social Security Benefits Values are reported in US dollars. The vertical dotted line in all 3 panels indicates the year in which the fracture occurred. Shaded areas indicate 95% CIs.

Postoperative infections were associated with a significant reduction in the annual household incomes of patients with fracture from 1 through 6 years after injury (Figure 1; Table 2). Within 1 year of injury, postoperative infections were associated with a $3160 loss (95% CI, −$5141 to −$1178; *P* = .002) in household incomes. In the 6 years after the fracture, a postoperative infection reduced patients’ household incomes by $6080 per year (95% CI, −$12 114 to −$47; *P* = .048). Postoperative infections after a fracture were associated with a 56.5% relative decrease (95% CI, −93.1% to −19.9%; *P* < .001) in household earnings within 2 years of injury and a 53.4% relative decrease (95% CI, −85.6% to −21.2%; *P* < .001) in household earnings within 5 years of injury.

**Table 2.** Adjusted Annual Mean Differences in Household Income, Individual Income, and Social Security Benefits Estimated in Models Censored From 1 to 6 Years After Injury

Outcome, time frame	Mean (SE), $		Adjusted annual mean difference (95% CI), $	*P* value
	Postoperative infection group	Uninfected control group		
**Household income**				
Before injury^a^	27 379 (2339)	30 617 (849)	NA	NA
At 1 y	18 516 (2338)	25 390 (575)	−3160 (−5141 to −1178)	.002
At 2 y	20 156 (2426)	26 022 (622)	−6368 (−12 252 to −483)	.03
At 3 y	17 123 (2009)	24 801 (720)	−6790 (−12 262 to −1317)	.02
At 4 y	16 797 (1999)	22 791 (795)	−6582 (−12 020 to −1143)	.02
At 5 y	14 912 (2300)	21 239 (1615)	−6520 (−12 502 to −538)	.03
At 6 y	14 022 (1811)	17 953 (631)	−6080 (−12 114 to −47)	.048
**Individual income**				
Before injury^a^	20 846 (2007)	19 137 (579)	NA	NA
At 1 y	13 695 (1805)	18 054 (416)	−4359 (−8727 to 10)	.05
At 2 y	14 042 (1705)	18 208 (412)	−4274 (−8348 to −200)	.04
At 3 y	13 210 (1741)	17 164 (408)	−4167 (−8147 to −187)	.04
At 4 y	12 333 (1609)	16 112 (411)	−4067 (−7975 to −159)	.04
At 5 y	12 157 (2113)	15 620 (1557)	−3972 (−8367 to 423)	.08
At 6 y	10 265 (1455)	13 565 (805)	−3844 (−8430 to 743)	.10
**Social Security benefits**				
Before injury^a^	152 (87)	960 (47)	NA	NA
At 1 y	1138 (270)	1289 (54)	−288 (−477 to −98)	.002
At 2 y	1721 (341)	1445 (58)	56 (−459 to 571)	.83
At 3 y	1789 (350)	1470 (60)	134 (−374 to 641)	.61
At 4 y	1574 (297)	1363 (58)	150 (−344 to 643)	.55
At 5 y	1525 (291)	1243 (55)	174 (−301 to 649)	.48
At 6 y	1480 (283)	1118 (52)	203 (−250 to 656)	.38

Abbreviation: NA, not applicable.

^a^ Preinjury levels were based on the tax year prior to injury.

**Figure 2.**
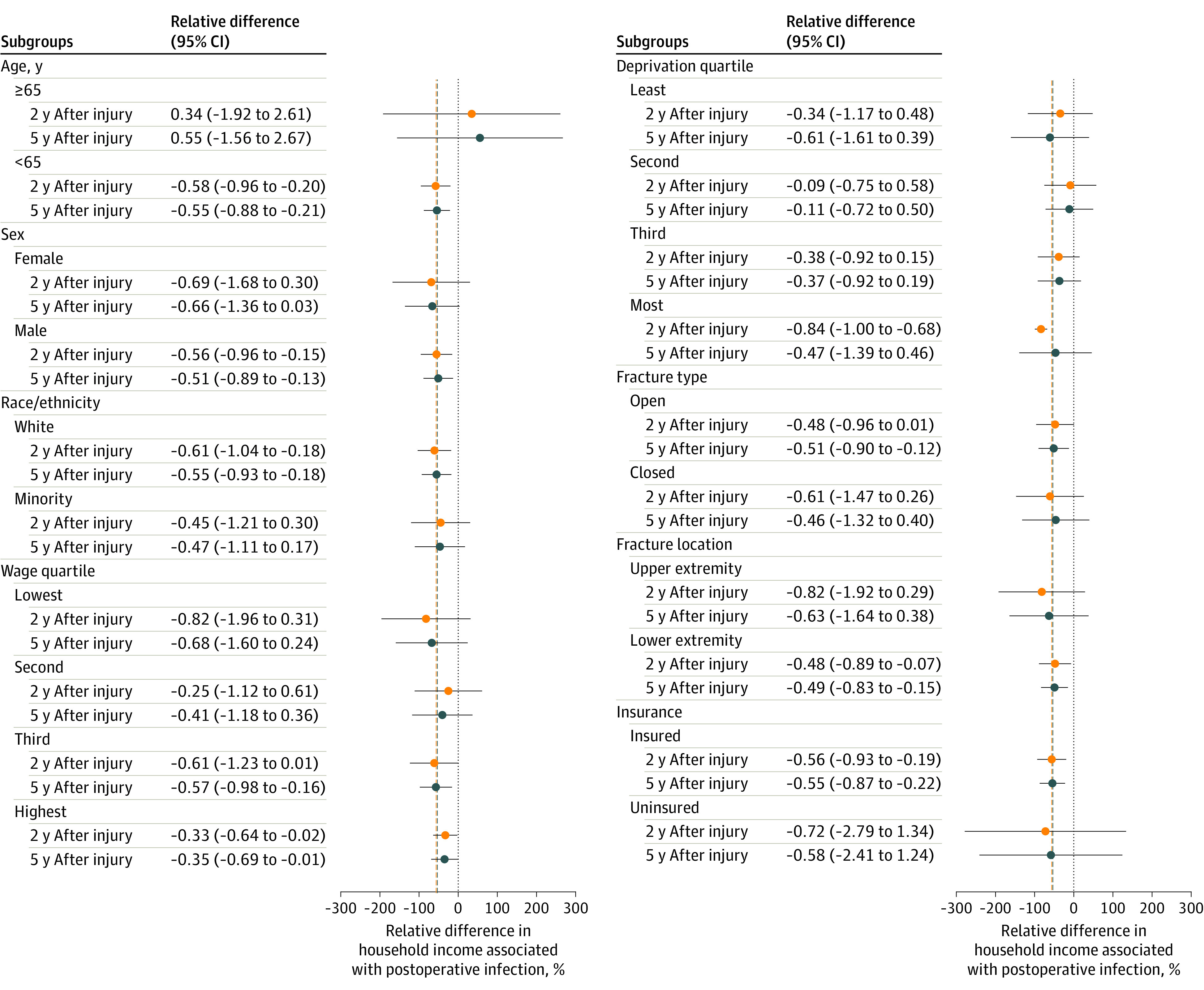
Relative Household Income Associated With a Postoperative Infection Within Subgroups and Evaluated at 2 and 5 Years After Fracture The dashed vertical lines represent sample mean estimates of the relative change in household income associated with a postoperative infection at 2 years and 5 years after fracture. The median incomes for the preinjury wage quartiles are as follows: lowest quartile, $0; second quartile, $5257; third quartile, $31 686; and highest quartile, $91 749.

The relative associations of a postoperative infection with household incomes varied based on the patient’s preinjury income quartile and neighborhood deprivation quartile (Figure 2). Specifically, the relative change in household income associated with a postoperative infection was significantly greater for patients in the lowest preinjury income quartile (difference, −82.4%; 95% CI, −196.2% to 31.3%) than for those in the highest preinjury income quartile (difference, −33.1%; 95% CI, −64.0% to −2.3%) within 2 years (*P* = .001) and 5 years of injury (lowest preinjury income quartile: difference, −67.7%; 95% CI, −159.6% to 24.2%; and highest preinjury income quartile: difference, −35.1%; 95% CI, −69.2% to −0.9%; *P* = .04). Second, patients in the lowest neighborhood deprivation quartile had a significantly greater loss in household income associated with a postoperative infection within 2 years of injury (difference, −83.7%; 95% CI, −99.5% to −67.9%) than patients living in the highest neighborhood deprivation quartile (difference, −34.4%; 95% CI, −117.3% to 48.3%; *P* = .03 for interaction).

### Individual Earnings

Postoperative infections were associated with reduced annual individual incomes by approximately $4000 for up to 6 years after injury (Figure 1; Table 2). However, the reductions in annual individual income associated with a postoperative infection were only statistically significant within 2, 3, and 4 years after the injury.

### Social Security and Unemployment Benefits

Postoperative infections were associated with a lower amount of Social Security benefits (difference, −$288; 95% CI, −$477 to −$98; *P* = .002) in the year after injury (Figure 1; Table 2). In the following years, we did not observe a significant association between a postoperative infection and the value of Social Security benefits received. However, a postoperative infection was associated with increased odds of receiving Social Security benefits at 2 years after fracture (OR, 1.74; 95% CI, 1.51-2.02; *P* < .001) and 5 years after injury (OR, 1.45; 95% CI, 1.25-1.68; *P* < .001) (eFigure 3 in the Supplement). Postoperative infections were not associated with receiving unemployment insurance at 2 years (OR, 0.94; 95% CI, 0.72-1.21; *P* = .62) but were associated with a 3-fold increase in the odds of receiving unemployment insurance at 5 years after injury (OR, 3.45; 95% CI, 2.82-4.23; *P* < .001) (eFigure 4 in the Supplement).

**Figure 3.**
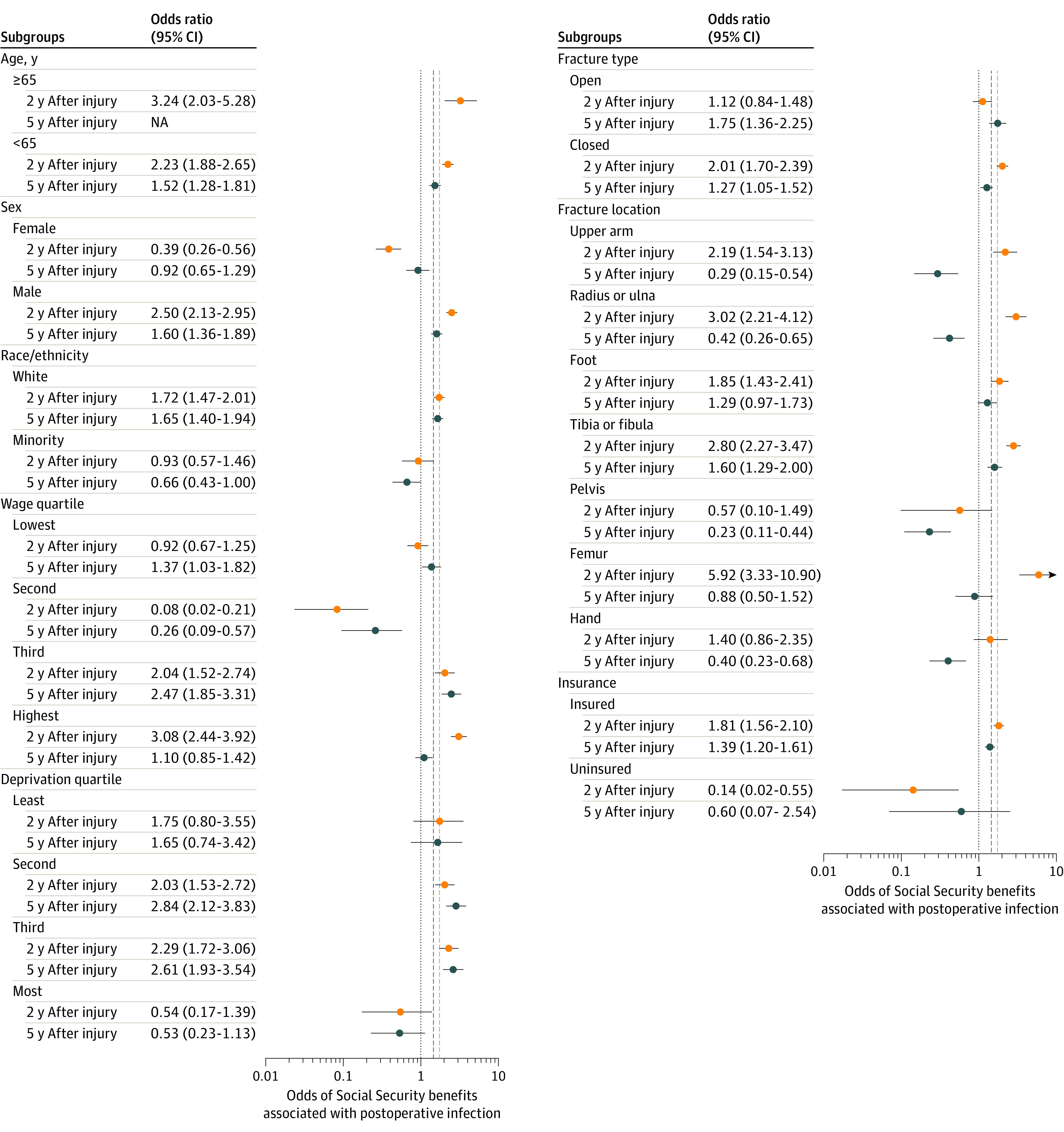
Odds of Receiving Social Security Benefits Associated With a Postoperative Infection Within Subgroups and Evaluated at 2 and 5 Years After Fracture The dashed vertical lines represent sample mean odds of receiving Social Security benefits associated with a postoperative infection at 2 years and 5 years after fracture. All postoperative infections occurred in patients 65 years of age or older at the time of injury who reported receiving Social Security benefits 5 years after their injury. The median incomes for the preinjury wage quartiles are as follows: lowest quartile, $0; second quartile, $5257; third quartile, $31 686; and highest quartile, $91 749. NA indicates not applicable.

The odds of receiving Social Security benefits after a postoperative infection varied by sex, race/ethnicity, fracture severity, preinjury income quartile, and neighborhood deprivation quartile (Figure 3). Male patients had greater odds of receiving Social Security benefits after a postoperative infection than female patients at 2 years (male patients: OR, 2.50; 95% CI, 2.13-2.95; female patients: OR, 0.39; 95% CI, 0.26-0.56) and 5 years after injury (male patients: OR, 1.60; 95% CI, 1.36-1.89; female patients: OR, 0.92; 95% CI, 0.65-1.29) (*P* < .001). White patients were more likely to receive Social Security benefits after a postoperative infection than were minority patients 2 years after injury (White patients: OR, 1.72; 95% CI, 1.47-2.01; minority patients: OR, 0.93; 95% CI, 0.57-1.46) and 5 years after injury (White patients: OR, 1.65; 95% CI, 0.40-1.94; minority patients: OR, 0.66; 95% CI, 0.43-1.00) (*P* < .001). Patients with closed fractures who sustained a postoperative infection were more likely to receive Social Security benefits within 2 years of injury (OR, 2.01; 95% CI, 1.70-2.39) than patients with open fractures who sustained a postoperative infection (OR, 1.12; 95% CI, 0.84-1.48) (*P* = .03). Conversely, patients with open fractures who developed a postoperative infection had greater odds of receiving Social Security benefits 5 years after injury (OR, 1.75; 95% CI, 1.36-2.25) compared with patients with closed fractures who developed a postoperative infection (OR, 1.27; 95% CI, 1.05-1.52) (*P* = .03). Two years after injury, patients in the highest (OR, 3.08; 95% CI, 2.44-3.92) and second-highest preinjury income quartiles (OR, 2.04; 95% CI, 1.52-2.74) had greater odds of receiving Social Security benefits after a postoperative infection than patients in the lowest (OR, 0.92; 95% CI, 0.67-1.25) and second-lowest income quartiles (OR, 0.08; 95% CI, 0.02-0.21) (*P* < .001). Five years after injury, patients in the lowest (OR, 1.37; 95% CI, 1.03-1.82) and second-highest preinjury income quartiles (OR, 2.47; 95% CI, 1.85-3.31) had greater odds of receiving Social Security benefits after a postoperative infection than patients in the highest income quartile (OR, 1.10; 95% CI, 0.85-1.42) (*P* < .001). Among patients living in neighborhoods with levels of deprivation in the middle quartiles, a postoperative infection doubled the odds of receiving Social Security benefits (second lowest quartile: OR, 2.03; 95% CI, 1.53-2.72; third lowest quartile: OR, 2.29; 95% CI, 1.72-3.06; *P* < .001) 2 years after injury.

### Catastrophic Wage Loss

Postoperative infections were associated with a 6.6% increase (95% CI, 4.9%-8.3%; *P* < .001) in the risk of catastrophic wage loss within 2 years of the fracture (eTable 3 in the Supplement). This estimate was robust when we increased the postinjury window to 5 years. When we restricted the definition of catastrophic wage loss to a 75% reduction in annual wage earnings, a postoperative infection increased the risk of catastrophic wage loss by 7.5% (95% CI, 6.1%-9.0%; *P* < .001). When we changed the definition of catastrophic wage loss to a 25% loss in mean wages, a postoperative infection was associated with a 4.3% increase in the risk of catastrophic wage loss (95% CI, 2.3%-6.2%; *P* < .001).

## Discussion

Our findings suggest that among patients with a fracture, a postoperative infection is associated with a $6080 reduction in annual household income. The income loss associated with postoperative infections represents approximately 20% of preinjury earnings, and the income loss persisted for at least 6 years after injury. Postoperative infections also increased the risk of catastrophic wage loss within 2 years of injury by approximately 7% and increased the odds of receiving Social Security benefits by 45%. However, incurring a postoperative infection was not associated with an increase in the amount of Social Security benefits received. Patients in the lowest preinjury income quartile experienced the greatest relative income loss yet had decreased odds of receiving Social Security benefits within 2 years of injury.

Clinical interventions and hospital policies to reduce hospital-associated infections are considered to be essential, but the economic impact of these infections remains understudied. Prior research suggests that the mean hospitalization costs in the US to treat a postoperative infection in a patient with a fracture exceed $20 000.^33,34^ The present value of $6000 in lost annual income over 6 years, discounted with a 2% inflation rate, is $34 281. Approximately 1 million patients sustain an operatively treated fracture in the US each year.^35^ Assuming that 3.5% of patients with a fracture have a postoperative infection, as observed in our study, the lost earnings associated with these complications would exceed $1 billion per year. Reducing the incidence of postoperative infections in patients with fracture by 1% would prevent more than $300 million in lost earnings annually.

Alternative payment models to incentivize patient safety, such as the Centers for Medicare & Medicaid Services’ Hospital Readmissions Reduction Program, have demonstrated positive outcomes.^36,37,38^ However, current readmission penalties are based on treating costs and fail to account for the indirect costs measured in our study. Furthermore, most current reimbursement programs do not apply to patients with fracture, and most postoperative infections in patients with fracture occur beyond the 30-day penalty window, implying that the treating institution’s patient safety responsibility should be extended.^39^

The costs of preventing postoperative infections are diffused within and between hospitals. The benefits of patient safety are also diffused at the hospital level but concentrated at the patient level. Circumstances with diffused costs and concentrated benefits at the individual patient level are typically favorable for advancing policy.^40^ However, the low socioeconomic status of many trauma patients suggests that this patient population has limited political capital to direct additional resources toward patient safety.

The preinjury household income levels of the sample were less than half the state median household income level. This difference highlights the profound socioeconomic deprivation faced by many patients with orthopedic trauma prior to their injury. The low reported earnings imply that many patients with fracture were without formal employment prior to their injury.^41^ This lack of workforce participation would negate the patient’s eligibility to obtain sick leave benefits from a workplace, unemployment insurance, and Social Security Disability Insurance. The early access to Social Security benefits in this study was realized by patients who were male and White and were in the top income quartile. This allocation inequity questions the distributional fairness of social insurance programs. The distributions observed in the study provide further evidence of limited social welfare for historically disenfranchised subpopulations.

Despite the frequency and associated socioeconomic impacts, surgical site infections that occur after fracture surgery are not currently included in the National Healthcare Safety Network Surgical Care Improvement Project. Therefore, they are not captured in the Agency for Healthcare Research and Quality’s National Scorecard on Hospital-Acquired Conditions.^10^ Given the profound societal costs, surgical site infections that occur after fracture surgery should be included in this national health care quality surveillance program.^42,43^ The financial implications of postoperative infections for patients should be included in evaluating infection prevention interventions and should be used to assess the marginal value of public investments in patient safety.^44,45^ Finally, the study highlights the disconnect between health care and social insurance programs, supporting the need for the further integration of these 2 domains.^46^

### Limitations

The study had several limitations. Postoperative infections were retrospectively identified in patients with fracture by using *CPT* codes; therefore, the potential for misclassification exists. However, a certified infection preventionist independently reviewed the medical records of each suspected case. The economic associations were estimated solely based on tax-reported earnings. We observed greater attrition in tax filing for patients with postoperative infection and cannot completely discount the endogenous associations of nonfiling in both groups. Prior studies suggest that an annual income lower than the Internal Review Service age-dependent thresholds of approximately $20 000 is the most common reason for nonfiling.^47^ Furthermore, data on incurred debt and consumption associations were not included in our estimates. The inclusion of these associations would likely amplify the societal costs of postoperative infections further. The study data did not report the components of Social Security income separately; therefore, we were unable to assess the association between postoperative infections and means-tested (eg, Supplemental Security Income) vs event-conditioned (eg, Disability Insurance or Retirement Income) Social Security programs. We did not have data on preinjury occupations, which would have likely explained some of the observed variances. The study data were collected from a single trauma center, and tax records were obtained from a single state, limiting the generalizability of the findings. However, obtaining a precise estimate of the financial consequences of a postoperative infection using tax data represents a substantial advancement compared with prior estimates.^5,6,7,33^ Furthermore, the analytical framework for calculating the economic associations of postoperative infections is transferrable to other health conditions and underscores the value of expanded use of administrative data for health outcomes research and the improvement of the safety of health care delivery.

## Conclusions

This study used a novel approach of linking hospital data with tax records to evaluate the association between a postoperative infection and long-term income among patients with surgically treated fractures in Maryland. Our study suggests that an operatively treated fracture is associated with substantially reduced patient income.^15^ The data from this study also suggest that postoperative infections may have a significant and sustained effect on patient income that is in excess of the loss associated with the fracture. The current social insurance mechanisms may not offset the decreased earnings. Given the long-term economic associations of a postoperative infection, health care professionals should be incentivized to continue seeking high-return investments in patient safety.^8^ Substantial economic benefits can be achieved through incremental improvements in infection prevention.

## Notes

Supplement. **eFigure 1.** Flow Chart **eFigure 2.** Attrition in Tax Filing **eFigure 3.** Proportion of Patients Receiving Social Security Benefits **eFigure 4.** Proportion of Patients Receiving Unemployment Insurance **eTable 1.** *Current Procedural Terminology* (CPT), *International Classification of Diseases, 9^th^ Revision* (ICD-9), and *10^th^ Revision* (ICD-10) Codes Used to Identify Patients With a Suspected Postoperative Infection **eTable 2.** Summary of Missing Data and Methods of Imputations **eTable 3.** Sensitivity Analysis for Catastrophic Wage Loss After a Postoperative Infection
