# Supplementary material for: Association of Postoperative Infections After Fractures With Long-term Income Among Adults
Source: JAMA Netw Open. 2021 Apr 19;4(4):e216673. doi: 10.1001/jamanetworkopen.2021.6673 (PMC8056283; doi:10.1001/jamanetworkopen.2021.6673)
Supplement: Supplement. — eFigure 1. Flow Chart eFigure 2. Attrition in Tax Filing eFigure 3. Proportion of Patients Receiving Social Security Benefits eFigure 4. Proportion of Patients Receiving Unemployment Insurance eTable 1. Current Procedural Terminology (CPT), International Classification of Diseases, 9th Revision (ICD-9), and 10th Revision (ICD-10) Codes Used to Identify Patients With a Suspected Postoperative Infection eTable 2. Summary of Missing Data and Methods of Imputations eTable 3. Sensitivity Analysis for Catastrophic Wage Loss After a Postoperative Infection [file jamanetwopen-e216673-s001.pdf]

# Supplementary Online Content

O'Hara NN, Mullins CD, Slobogean GP, Harris AD, Kringos DS, Klazinga NS. Association of postoperative infections after fractures with long-term income among adults. *JAMA Netw Open.* 2021;4(4):e216673. doi:10.1001/jamanetworkopen.2021.6673

**eFigure 1.** Flow Chart
**eFigure 2.** Attrition in Tax Filing
**eFigure 3.** Proportion of Patients Receiving Social Security Benefits
**eFigure 4.** Proportion of Patients Receiving Unemployment Insurance
**eTable 1.** *Current Procedural Terminology* (CPT), *International Classification of Diseases, 9th Revision* (ICD-9), and *10th Revision* (ICD-10) Codes Used to Identify Patients With a Suspected Postoperative Infection
**eTable 2.** Summary of Missing Data and Methods of Imputations
**eTable 3.** Sensitivity Analysis for Catastrophic Wage Loss After a Postoperative Infection

This supplementary material has been provided by the authors to give readers additional information about their work.

© 2021 O'Hara NN et al. *JAMA Network Open.*

**eFigure 1.** Flow Chart

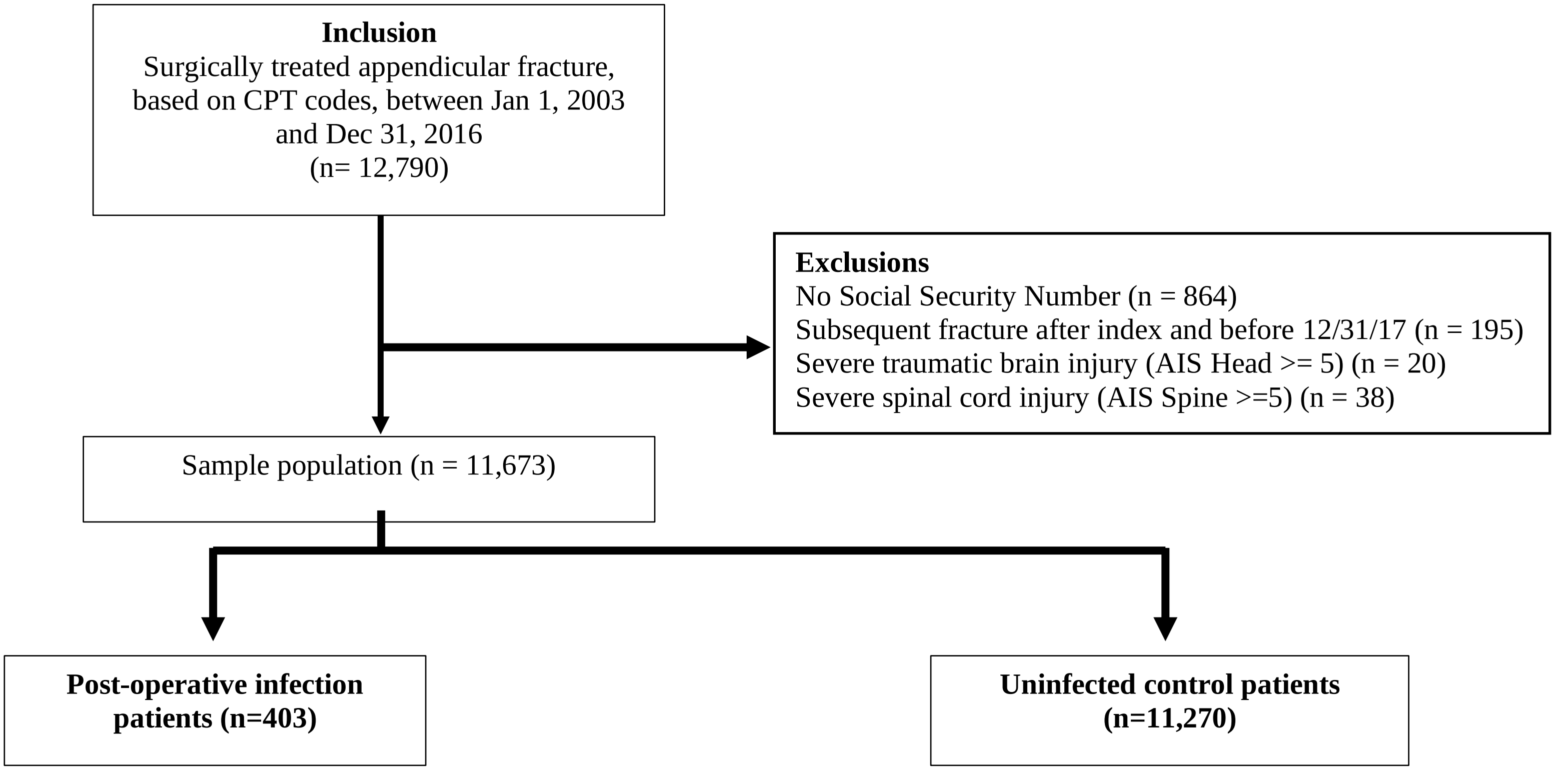


© 2021 O'Hara NN et al. *JAMA Network Open.*

**eFigure 2.** Attrition in Tax Filing

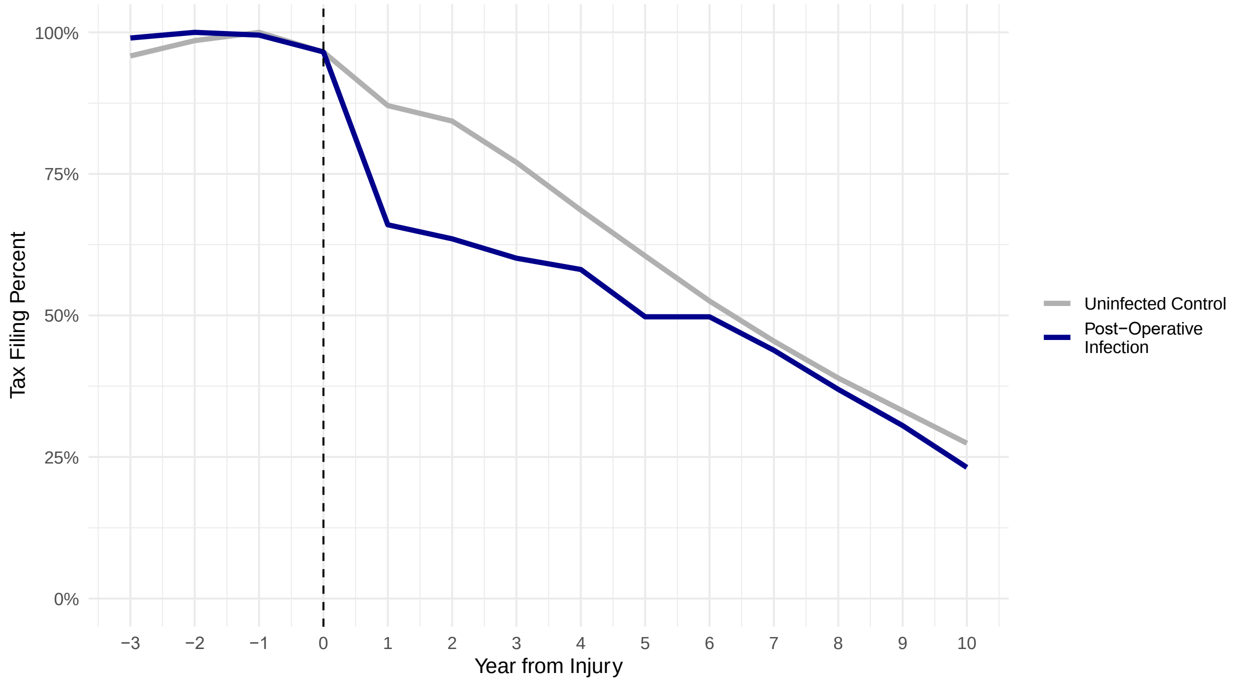


© 2021 O'Hara NN et al. *JAMA Network Open.*

**eFigure 3.** Proportion of Patients Receiving Social Security Benefits

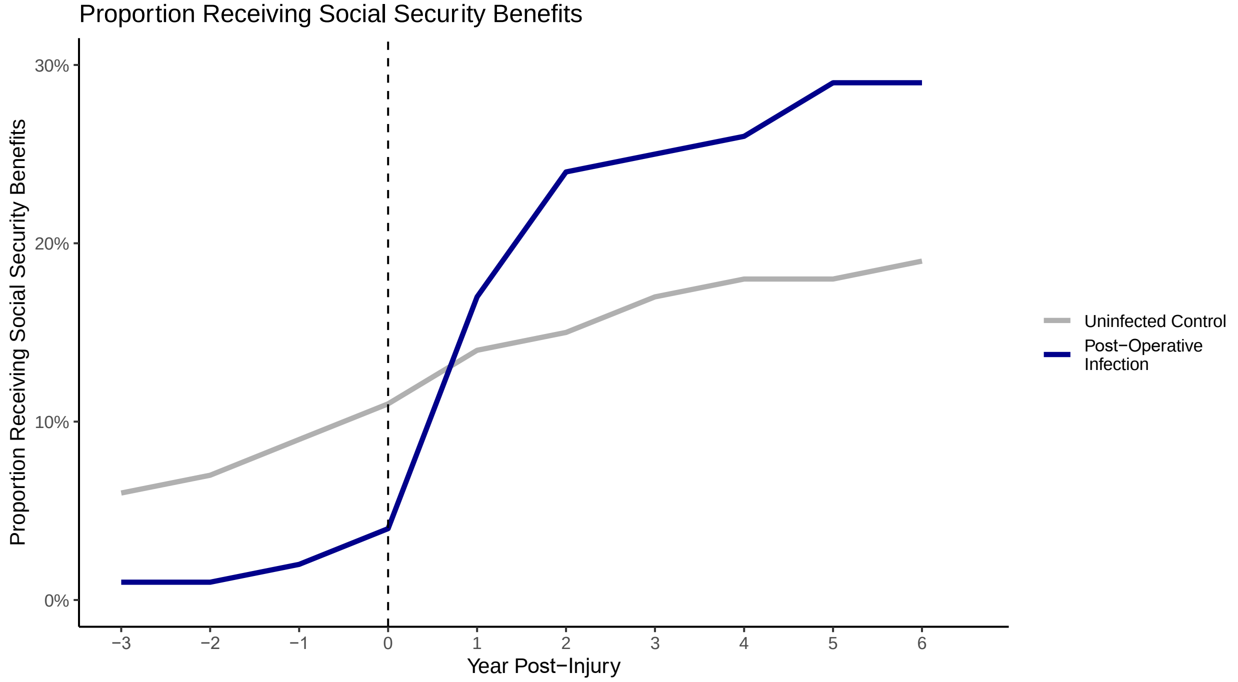


© 2021 O'Hara NN et al. *JAMA Network Open.*

**eFigure 4.** Proportion of Patients Receiving Unemployment Insurance

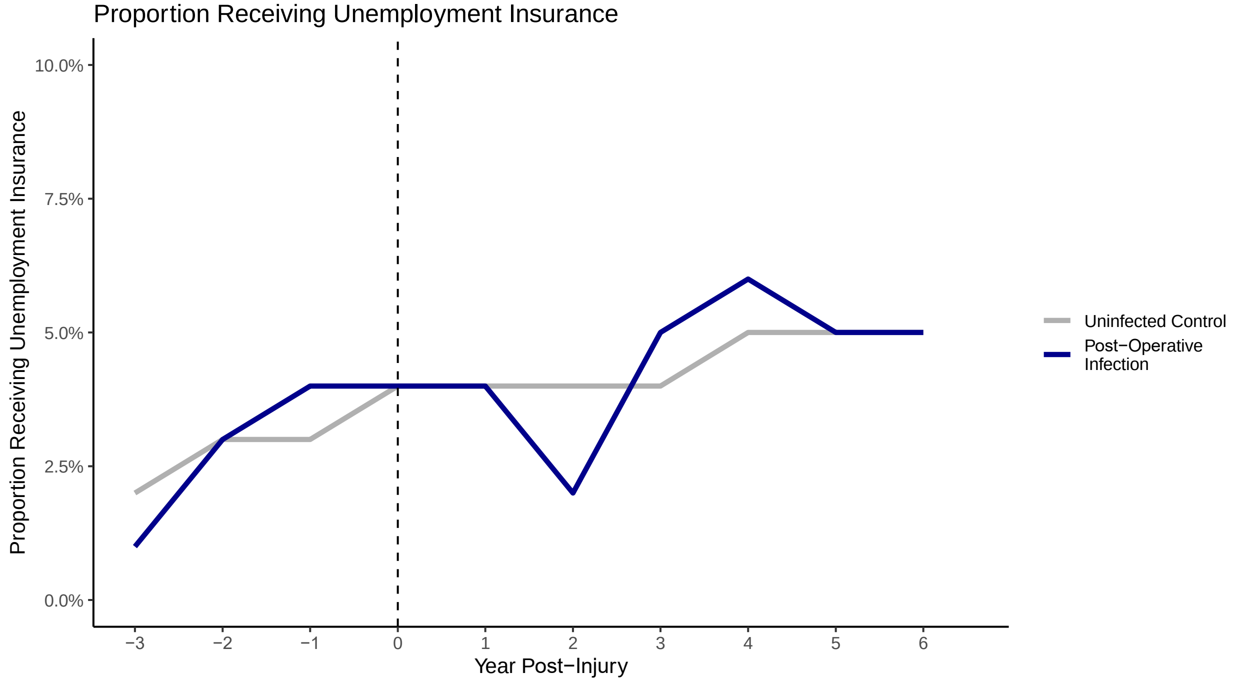


© 2021 O'Hara NN et al. *JAMA Network Open.*

**eTable 1.** *Current Procedural Terminology* (CPT), *International Classification of Diseases, 9th Revision* (ICD-9), and *10th Revision* (ICD-10) Codes Used to Identify Patients With a Suspected Postoperative Infection

| Code | Description |
|---|---|
| *ICD-9* | |
| 730.2 | Unspecified osteomyelitis |
| 730.20 | Unspecified osteomyelitis site unspecified |
| 730.21 | Unspecified osteomyelitis involving shoulder region |
| 730.22 | Unspecified osteomyelitis involving upper arm |
| 730.23 | Unspecified osteomyelitis involving forearm |
| 730.24 | Unspecified osteomyelitis involving hand |
| 730.25 | Unspecified osteomyelitis involving pelvic region and thigh |
| 730.26 | Unspecified osteomyelitis involving lower leg |
| 730.27 | Unspecified osteomyelitis involving ankle and foot |
| 730.28 | Unspecified osteomyelitis involving other specified sites |
| 730.29 | Unspecified osteomyelitis involving multiple sites |
| 998.5 | Postoperative infection not elsewhere classified |
| 996.67 | Infection and inflammatory reaction due to other internal orthopedic device implant and graft |
| | |
| *ICD-10* | |
| T81.3 | Disruption of wound, not elsewhere classified |
| T81.30 | Disruption of wound, unspecified |
| T81.31 | Disruption of external operation (surgical) wound, not elsewhere classified |
| T81.32 | Disruption of internal operation (surgical) wound, not elsewhere classified |
| T81.33 | Disruption of traumatic injury wound repair |
| T81.4 | Code for Infection following a procedure |
| T81.40 | Code for Infection following a procedure, unspecified |
| T81.41 | Infection following a procedure, superficial incisional surgical site |
| T81.42 | Infection following a procedure, deep incisional surgical site |
| T81.43 | Infection following a procedure, organ and space surgical site |
| T81.44 | Sepsis following a procedure |
| T81.49 | Infection following a procedure, other surgical site |
| T84.5 | Infection and inflammatory reaction due to internal joint prosthesis |
| T84.51 | Infection and inflammatory reaction due to internal right hip prosthesis |
| T84.52 | Infection and inflammatory reaction due to internal left hip prosthesis |
| T84.53 | Infection and inflammatory reaction due to internal right knee prosthesis |
| T84.54 | Infection and inflammatory reaction due to internal left knee prosthesis |
| T84.59 | Infection and inflammatory reaction due to other internal joint prosthesis |
| T84.6 | Infection and inflammatory reaction due to internal fixation device |
| T84.60 | Infection and inflammatory reaction due to internal fixation device of unspecified site |
| T84.61 | Infection and inflammatory reaction due to internal fixation device of arm |
| T84.62 | Infection and inflammatory reaction due to internal fixation device of leg |
| T84.63 | Code for Infection and inflammatory reaction due to internal fixation device of spine |
| T84.69 | Infection and inflammatory reaction due to internal fixation device of other site |
| T84.7 | Infection and inflammatory reaction due to other internal orthopedic prosthetic devices, implants and grafts |
| T84.7XXA | Infection and inflammatory reaction due to other internal orthopedic prosthetic devices, implants and grafts, initial encounter |

© 2021 O'Hara NN et al. *JAMA Network Open.*

| | |
|---|---|
| T84.7XXD | Infection and inflammatory reaction due to other internal orthopedic prosthetic devices, implants and grafts, initial encounter |
| T84.7XXS | Infection and inflammatory reaction due to other internal orthopedic prosthetic devices, implants and grafts, sequela |
| M86.9 | Osteomyelitis, unspecified |
| | |
| *CPT Codes* | |
| 11011 | Debridement including removal of foreign material associated with open fracture(s) and/or dislocation(s) |
| 11012 | Debridement including removal of foreign material associated with open fracture(s) and/or dislocation(s) |
| 11043 | Under Debridement Procedures on the Skin |
| 11044 | Under Debridement Procedures on the Skin |
| 10180 | Under Incision and Drainage Procedures on the Skin, Subcutaneous and Accessory Structures |
| 27360 | Under Excision Procedures on the Femur (Thigh Region) and Knee Joint |
| 27640 | Under Excision Procedures on the Leg (Tibia and Fibula) and Ankle Joint |
| 28120 | Under Excision Procedures on the Foot and Toes |
| 28122 | Under Excision Procedures on the Foot and Toes |

© 2021 O'Hara NN et al. *JAMA Network Open.*

**eTable 2.** Summary of Missing Data and Methods of Imputations

| **Covariate** | **Missing Data (n=11,673)** | **Imputation Method** |
|---|---|---|
| **Age,** n (%) | 0 (0%) | NA |
| **Male,** n (%) | 0 (0%) | NA |
| **Race,** n (%) | 133 (1.1%) | Polytomous logistic regression |
| **Area Deprivation Index,** n (%) | 2214 (19.0%) | Predictive mean matching |
| **Mechanism of injury**, n (%) | 0 (0%) | NA |
| **Injury type**, n (%) | 0 (0%) | NA |
| **Glasgow Comma Score**, n (%) | 0 (0%) | NA |
| **Health insurance**, n (%) | 0 (0%) | NA |
| **Abbreviated Injury Scale** | | NA |
| Lower extremity, n (%) | 0 (0%) | NA |
| Upper extremity, n (%) | 0 (0%) | NA |
| Abdominal, n (%) | 0 (0%) | NA |
| Face, n (%) | 0 (0%) | NA |
| Head, n (%) | 0 (0%) | NA |
| Neck, n (%) | 0 (0%) | NA |
| Spine, n (%) | 0 (0%) | NA |
| Chest, n (%) | 0 (0%) | NA |
| **Comorbidities** | | NA |
| Alcohol dependence, n (%) | 0 (0%) | NA |
| Cancer, n (%) | 0 (0%) | NA |
| Depression, n (%) | 0 (0%) | NA |
| Diabetes, n (%) | 0 (0%) | NA |
| IV drug use, n (%) | 0 (0%) | NA |
| Non-IV drug use, n (%) | 0 (0%) | NA |
| Hypertension, n (%) | 0 (0%) | NA |
| Tobacco use, n (%) | 0 (0%) | NA |
| **Fracture location**, n (%) | 0 (0%) | NA |
| **Open fracture**, n (%) | 0 (0%) | NA |

© 2021 O'Hara NN et al. *JAMA Network Open.*

**eTable 3.** Sensitivity Analysis for Catastrophic Wage Loss After a Postoperative Infection

| **Level of Catastrophic Wage Loss** | **Years Post-Injury** | **Post-Operative Infection (n=160)** | **Unexposed Control (n=4597)** | **Adjusted Mean Difference (95% CI)** | ***P* Value** |
|---|---|---|---|---|---|
| **50%** | **2 years** | **46 (28.8%)** | **833 (18.1%)** | **6.6% (4.9 to 8.3)** | **<0.001** |
| | 5 years | 53 (31.5%) | 861 (18.1%) | 6.8% (5.1 to 8.6) | <0.001 |
| | | | | | |
| 25% | 2 years | 68 (42.5%) | 1384 (30.1%) | 4.3% (2.3 to 6.2) | <0.001 |
| | 5 years | 70 (41.7%) | 1382 (29.1%) | 4.4% (2.5 to 6.3) | <0.001 |
| | | | | | |
| 75% | 2 years | 23 (14.4%) | 456 (9.9%) | 7.5% (6.1 to 9.0) | <0.001 |
| | 5 years | 27 (16.1%) | 490 (10.3%) | 6.4% (5.0 to 7.8) | <0.001 |

**Note:** Mean differences were adjusted using inverse probability of treatment weighting.

© 2021 O'Hara NN et al. *JAMA Network Open.*
